# Changes in the lifetime prevalence of suicidal feelings and thoughts among Norwegian doctors from 2000 to 2010: a longitudinal study based on national samples

**DOI:** 10.1186/1471-244X-13-322

**Published:** 2013-11-28

**Authors:** Judith Rosta, Olaf G Aasland

**Affiliations:** 1 Institute for Studies of Medical Profession (LEFO), Oslo, Norway; 2 Institute of Health and Society, Department of Health Management and Health Economics, University of Oslo, Oslo, Norway

**Keywords:** Suicidal feelings, Doctors, Norway, Longitudinal study

## Abstract

**Background:**

Thinking about suicide is an indicator of suicide risk. Suicide rates are higher among doctors than in the population. The main aims of this study are to describe the changes in the lifetime prevalence of suicidal feelings from 2000 to 2010 and the possible predictors of serious suicidal thoughts in 2010 among Norwegian doctors. Differences in lifetime prevalence of suicidal feelings between Norwegian doctors in 2010 and German doctors in 2006 will be also described.

**Methods:**

Longitudinal and cross-sectional study based on questionnaire data from 2000 and 2010, including approximately 1,600 Norwegian doctors. In Germany, cross-sectional study based on questionnaire data from 2006 among a sample of 3,295 doctors. The main outcome measures were the lifetime prevalence of suicidal feelings (felt life was not worth living, wished own death, had thoughts of taking own life).

**Results:**

The prevalences in 2000 and 2010 of ever had feelings of life not worth living were 48 (44 to 52) % and 45 (41 to 49) %, of ever wished own death 27 (23 to 30) % and 23 (20 to 26) %, and of ever had thoughts of taking own life 29 (16 to 33) % and 24 (21 to 27) %. Paired t-tests among those who responded both in 2000 and 2010 show significant reductions for felt life not worth living (t = −3.4; p = 0.001), wished own death (t = −3.1; p = 0.002) and had thoughts of taking own life (t = −3.5; p < 0.0001). In 2010, significant predictors of serious suicidal thoughts in a multivariate model were low subjective well-being (OR 0.68; 95% CI 0.52-0.90), poor or average self-rated health (2.36; 1.25-4.45) and high psychosocial work stress (1.92; 1.06-3.46), controlled for age, gender, speciality and job satisfaction. Norwegian doctors in 2010 compared with their German counterparts in 2006 reported quite similar prevalences of suicidal feelings.

**Conclusions:**

Suicidal feelings among Norwegian doctors decreased from 2000 to 2010. Individual and work-related factors may to certain explain these findings. Compared with other professionals in Norway and doctors in Germany, Norwegian doctors showed no higher risk of suicidal thoughts.

## Background

Thinking about suicide is one of several factors that may increase the risk of suicide. Suicide mortality is higher among doctors than in the population, [1,2] and to lower the suicide rate in this group should in itself be an important responsibility for the profession. Regular screening for having ideas or thoughts on taking own life is in itself an important preventive measure on the individual as well as on the aggregate level [3]–[5].

The 2012 ICD-9-CM Diagnosis Code V62.84 describes *suicidal ideation* as “thoughts of or an unusual preoccupation with suicide”. Risk factors for different degrees of suicidal ideation range from having ideas on own death to making suicidal plans and attempting suicide [6]. Mental disorders, particularly depression, are well-known risk factors, but physical illnesses, family history or traumatic life events are also important [7]. Social factors are also present in suicide. Durkheim [8] found associations between suicide rate, social disintegration and dissatisfaction in relation to expectations in several European countries. Recent studies support the impact of social network (social capital) on population health outcomes [9], suicide mortality, [10] and also suicide ideation [11]. Thinking on own suicide can also be seen as an expression of a hopeless situation. The Norwegian University Hospital Ulleval reported that among all admissions due to self-inflicted poisoning 20% were suicide attempts, 20% accidents and 60% appeal reactions [12].

Thinking about suicide at one point in life is not uncommon, [13,14] but most persons with suicidal ideation do not proceed to action. However, in a recent collaborative study across seventeen countries, [14] the probability of ever making a suicide attempt was judged to be .29 among subjects with suicidal ideation, of which 60% occurred within the first year after ideation onset. Hence, suicidal ideation must be regarded as a short term risk factor for suicide [15].

Most studies of doctors’ suicidal ideation are based on cross-sectional data, and there is a need for longitudinal studies. A lifetime prevalence of suicidal ideation of 14.9% was reported by American surgeons in 2008, [16] and of 33.5% by Swedish and 21.4% by Italian female doctors working in University hospitals in 2005 [17]. In 2007, 11.2% of US medical students had experienced suicidal ideation over a twelve-month period [18]. One in four Finnish anaesthesiologists were identified as suicidal in 2005, [3] and 1.5% of female US doctors in 1998 reported a history of attempted suicide at some point over the lifetime [19].

In Norway, a study with data from 1993 showed that one in two doctors at one point in time felt that life was not worth living, one in three had sometime wished to be dead, and more than this had experienced suicidal ideation on one or more occasions [20]. Two other studies, one on police officers with data from 2000 [21] and another on operational ambulance personal with data from 2005 [22] suggest higher levels of suicidal ideation in doctors. A study of Norwegian medical school graduates from 1993 and 1994 showed stability in previous year’s suicidal thoughts from medical school and into the first postgraduate year [23].

There are no trend analyses on suicidal ideation in Norway. The Eurostat data from 1995 to 2010 show a decline in death by suicide in most European countries, including Norway [24]. A national study based on Data from The Causes-of-Death Registry of Statistics Norway from 1990 to 2006 found that the total suicide rate decreased by 26% [25]. Other studies with data from 1960 to 2000 report that doctors had higher suicide rates than the general population, [1] but the gap between the doctors and the general population seems to be slowly closing [2]. This reduction in suicide mortality may imply a contemporary reduction in suicidal ideation, also among doctors.

We have assessed suicidal feelings (feeling of life not worth living, suicidal ideation and suicidal thoughts) of Norwegian doctors in 2000 and 2010, and possible changes during this period. We also identify possible predictors of serious suicidal thoughts in 2010. Finally we compare the Norwegian data with data from a nation-wide survey of German doctors in 2006. International differences in doctors’ suicidal feelings are of interest. In 2006 a postal survey using the same instruments on suicidal feelings was carried out among doctors in Germany. It is therefore possible to perform a comparative study where data on suicidal feelings from doctors in two European countries can be reliably compared. Because the German sample includes hospital doctors only, the present analyses will be limited to this category.

## Methods

### Study design and sample

Since 1994 the Institute for Studies of the Medical Profession in Norway has regularly surveyed a representative cohort of about 1,270 active Norwegian doctors with postal questionnaires. The cohort was supplemented with approximately 400 randomly selected doctors in 2000, and with another 250 in 2008. Over the whole period, approximately 400 doctors have left the cohort due to death, retirement or voluntary withdrawal. The present study is based on data from 2000 and 2010.

### Ethics

The project is in compliance with the declaration of Helsinki on Ethical Principles for Medical Research Involving Human Subjects adopted by the World Medical Association (http://www.wma.net/en/30publications/10policies/b3/index.html). According to the Regional Committee for Medical Research Ethics, the study based on “Norwegian Physician Survey - A bi-annual prospective questionnaire survey to a representative sample of Norwegian physicians” is exempt from review in Norway, cf. §§ 4 of The Act. The project can be implemented without the approval by the Regional Committee for Medical Research Ethics (IRB 0000 1870). Additionally, approval for data protection of the bi-annual prospective survey among Norwegian doctors was obtained from the Norwegian Social Science Data Service (Reference 19521).

### Measurements

#### Suicidal feelings

Different instruments exist to measure suicidal ideation, suicidal thoughts, and suicidal feelings using one or several questions [4,21,23]. In our study, we used the Paykel’s Suicidal Feelings in the General Population questionnaire [6]. It contains five items with increasing seriousness from feelings that life is not worth living to actual suicidal attempts. We used all five items in 2000, but only items 1 to 3 in 2010, due to the low prevalence of positive answers on items 4 and 5.

The questions are:

(1) “Have you ever felt that life was not worth living?”

(2) “Have you ever wished you were dead (for instance, that you could get to sleep and not wake up)?”

(3) “Have you ever through of taking your own life, even if you would not really do it?”

(4) “Have you ever reached the point where you seriously considered taking your life, or perhaps made plans how you would go about doing it?

(5) “Have you ever made an attempt to take your life?”

Items 1 to 3 have four response alternatives: never, hardly ever, sometimes and often. Item 4 has six response alternatives: never, once, 2–3 times, 4–5 times, 6–9 times and 10 or more times, and item 5 has four response alternatives: never, once, twice and 3 or more times.

#### Terminology and categorisation of responses

In this study, suicidal feelings refer to the three first questions. In accordance with a previous study among young doctors, [23]*suicidal ideation* refers to question two, and *suicidal thoughts* to question three, s*uicidal planning* to question four and *suicidal attempt* to question five.

#### Potential predictors of serious suicidal thoughts

Numerous factors for doctors’ suicidal ideation and suicidal thoughts have been described in previous studies, for example mental and physical health, distress, personality traits, marital status, work conditions, medical specialty [17]–[20,23,26]. The present study could include the following items:

*Psychosocial stress at work* was measured in 2010 by the validated short form of the effort-reward questionnaire (ERI) [27]. It comprises four items from the effort scale (time pressure, interruptions/disturbances, responsibility and demanding in the job) and five items from the reward scale (remuneration, esteem/appreciation, career opportunities such promotion, job security). Estimations were given on a five point Likert scale. According to this model, work stress is rooted in a chronic mismatch between high efforts and low rewards. Hence, a ratio of the sum score of the effort items (nominator) relative to sum score of the reward items (adjusted for the number of items; denominator) greater than one indicates a high level of psychosocial work stress.

*Self-rated health* was measured in 2010 by the question “In general, would you say your health is: very good/ good/ not very good (average)/poor”. This item has been shown correlate with mortality risk, morbidity, and general health status [28].

A question on *subjective well-being* (or general life satisfaction) was used in 2010, and scored on the same Likert scale. This question reads as follows: “When you think about your life at the moment, would you say that by and large you are satisfied with life or are you mostly dissatisfied?” This one-item measure of subjective well-being was originally derived from the Norwegian HUNT-study [29] and is widely used in national and international surveys [29,30].

*Job satisfaction* was measured with the Warr, Cook and Wall Job satisfaction scale [31]. It includes ten items that scored on a Likert scale from 1 (very dissatisfied) to 7 (very satisfied). The items were added together into a composite mean job satisfaction scale (JSS) with possible values from 10 to 70. The items are: How satisfied are you with: the amount of responsibility you are given variation on work, your colleagues and fellow workers, your physical work conditions, your opportunities to use your skills, your overall job situation, the freedom to choose your own methods of working, the recognition you get for good achievements, your rate of pay, your work hours.

Background variables are *gender*, *age* and *medical specialty. Medical specialties*, of which there are 44 in Norway, are collapsed into six categories: *surgery* (including gastroenterologic-, child-, vascular-, orthopaedic-, thoracic-, maxillofacial-, neuro-, plastic-, urologic- and general surgery plus ophthalmology), *internal medicine* (including haematology, endocrinology, gastroenterology, geriatrics, cardiology, infectious diseases, pneumatology, nephrology, rheumatology, oncology, and general practice/family medicine), *anaesthesiology*, *gynaecology*, *psychiatry* (including child and adolescent psychiatry) and other or no specialty.

#### Data on German doctors

In Germany, a postal questionnaire on health and working conditions was sent from the German Hospital Institute to 3,295 German hospital doctors with no reminder in 2006. The data collection among German doctors was supported by The German Research Foundation. One of the author’s (JR) is the principal investigator and research leader of the national questionnaire survey of hospital doctors in Germany. JR has permission to use the data from this national survey in your study (Reference DFG/RO 2348/4-1, 5–1). Survey methods, data and sample are described in details elsewhere [32].

The questionnaire included comparable data on demographics, medical specialty and the first three items of the Paykel’s instrument on suicidal feelings. It is therefore possible to perform a comparative study where data from doctors in Germany and Norway on suicidal feelings can be reliably compared. Because the German sample includes hospital doctors only, the comparison will be limited to this category.

#### Analyses

In this study, we paid particular attention to the first three items of Paykel’s instrument on suicidal feelings. Group differences were tested with the χ^2^ or 95% confidence intervals for proportions. Changes over time were tested with paired t-tests, based on the response means when “never” was given the value 0, hardly ever = 1, sometimes = 2 and often = 3. Simultaneous effects were analysed through logistic regression models. The Predictive Analytics SoftWare Statistics (PASW), version 18 was used for the analyses.

## Results

### Sample characteristics

The response rate was 86% (1,385/1,616) in 2000 and 67% (1,014/1,520) in 2010. The number of respondents who answered all three questions on suicidal feelings was 1,253 in 2000 and 962 in 2010. 715 doctors answered both in 2000 and 2010.

Table 1 shows gender, age and job situation for all respondents in 2000 and 2010, as well for the 715 doctors who answered both in 2000 and 2010, and how they compare with the total Norwegian doctor workforce in 2000.

**Table 1:** Characteristics of the respondents for whom questions 1, 2 and 3 of Paykel’s instrument could be calculated in 2000 and 2010, compared with all active doctors in Norway in 2000

	**All active doctors in Norway**	**Doctors who responded to items 1 to 3 of Paykel’s instrument**		**Doctors who responded to items 1 to 3 of Paykel’s instrument at two points in time**	
	**2000**	**2000**	**2010**	**2000 and 2010**	
**All** (n)	14,950	1,253	962	715	
**Gender**, % (95% CI)					
Females	30.1	32.5 (29.9–35.1)	38.7 (35.6-41.8)	32.7 (29.3-36.1)	32.7 (29.3-36.1)
Males	69.9	67.5 (64.9-70.1)	61.3 (58.2-64.4)	67.3 (63.9-70.4)	67.3 (63.9-70.7)
Missing (n)	(−)	(−)	(−)	(−)	(−)
**Age in years**, mean (95% CI)					
All	45.0	45.4 (44.9-46.0)	49.3 (48.7-49.9)	42.6 (42.0-43.3)	52.6 (52.0-53.3)
Missing (n)	(−)	(−)	(−)	(−)	(−)
**Job situation**, % (95% CI)					
Hospital doctors	51.2	54.4 (51.5-57.3)	55.5 (52.3-58.7)	53.5 (49.7-57.3)	51.3 (47.6-55.0)
Private practice specialists	5.4	5.2 (3.9-6.5)	6.3 (4.8-7.8)	5.0 (3.3-6.7)	8.6 (6.5-10.7)
General practitioners	23.3	26.9 (24.3-29.5)	27.0 (24.2-29.8)	27.3 (23.9-30.7)	27.4 (24.1-30.7)
Others	20.1	13.5 (11.5-15.5)	11.1 (9.1-13.1)	14.3 (11.6-17.0)	12.6 (10.2-15.1)
Missing (n)	(−)	(107)	(11)	(49)	(8)

### Changes in the lifetime prevalence of suicidal feelings from 2000 to 2010

Table 2 shows the responses to the three first Paykel items in 2000 and 2010. There is a trend towards a lower prevalence of suicidal feelings. When we calculate the means based on assigning the value 0 for “never”, 1 for “hardly ever”, 2 for “sometimes” and 3 for “often”, paired t-tests among those who responded both in 2000 and 2010 show significant reductions for all items.

**Table 2:** Prevalence of suicidal feelings ever in 2000 and 2010 among all respondents, and those who responded at both points in time

	**2000 (N = 1,253)**	**2010 (N = 962)**	**Change from 2000 to 2010 (N = 715)**
	percent (95% CI)	percent (95% CI)	paired t-test^(a)^
**Felt life was not worth living**			
Never	52.3 (49.7–55.3)	54.9 (51.8–58.0)	mean diff = − 0.9
Hardly ever	28.7 (26.0–31.0)	31.7 (28.8–34.6)	t = −3.4
Sometimes	17.2 (15.0–19.2)	12.4 (10.1–14.3)	p = 0.001
Often	1.8 (1.1–2.7)	1.0 (0.4–1.6)	
**Wished own death**			
Never	72.9 (70.1–75.5)	76.8 (70.2–76.6)	mean diff = −0.7
Hardly ever	17.6 (15.5–19.7)	15.6 (13.3–17.9)	t = −3.1
Sometimes	8.9 (7.1–10.3)	7.6 (5.9–9.3)	p = 0.002
Often	0.6 (0.2–1.2)	-	
**Thoughts of taking own life**			
Never	69.2 (66.9–72.0)	74.6 (71.2–77.4)	mean diff = −0.8
Hardly ever	20.6 (18.2–22.6)	17.7 (15.3–20.1)	t = −3.5
Sometimes	9.2 (7.5–10.7)	7.4 (5.8–9.1)	p < 0.0001
Often	1.0 (0.5–1.7)	0.3 (0.1–0.7)	

^(a)^Paired t-test. Calculated with the following response values: never = 0, hardly ever = 1, sometimes = 2, often = 3.

In 2000, 9.5% (117/1,236) of the doctors reported suicidal planning (item 4 of Paykel’s instrument), and 1.3% (16/1,247) had attempted suicide (item 5 of Paykel’s instrument) on one or more occasions in lifetime. These questions were not included in the 2010 survey.

### Change in response patterns for suicidal thoughts from 2000 to 2010

**Figure 1:**
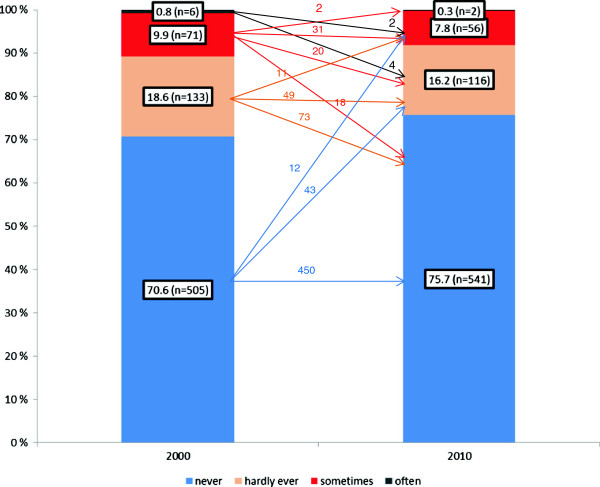
Change in response patterns for suicidal thoughts from 2000 to 2010 (n=715).

Figure 1 illustrates how the responses on suicidal thoughts changed from 2000 to 2010 among the 715 doctors who answered at both points in time. The prevalence of the response alternatives “often”, “sometimes” or “hardly ever” decreased, while the alternative “never” increased.

Of the 185 respondents who gave different answers, more changed to lower than to higher seriousness: from “often” to “sometimes”, from “sometimes” to “hardly ever” and “never”, and from “hardly ever” to “never”.

### Predictors of ever having serious suicidal thoughts among doctors in 2010

In 2010, 7.7% of the doctors reported that they had sometimes or often had suicidal thoughts (Table 2). Table 3 shows a multivariate logistic model with having ever had serious suicidal thoughts as response variable, age, gender, specialty group, job satisfaction, subjective well-being, self-rated health, psychosocial stress, and having answered or not in 2000 as possible simultaneous predictors. Subjective well-being, self-rated health and psychosocial work stress were all independent significant predictors of ever having serious suicidal thoughts. Gender, age, job satisfaction, speciality or having answered in 2000 showed no effect in the model.

**Table 3:** Logistic regression analyses with serious suicidal thought (dichotomized) as the response variable among Norwegian doctors who responded in 2010 and in both points in time

	**Serious suicidal thought (n = 890)**		
	**OR**	**95% CI**	**P**
Subjective well-being (sum score 1–7)	0.68	0.52-0.90	0.007
Poor or average self-rated health (vs. good or very good)	2.36	1.25-4.45	0.008
High levels of psychosocial work stress (vs. low levels)	1.92	1.06-3.46	0.031

Controlled for gender, age in years, medical specialty (surgery, internal medicine, anaesthesiology, gynaecology, psychiatry and other or no specialty), job satisfaction (sum score 10–70), responded in 2000 (vs. not responded in 2000).

### Comparison of suicidal feelings in Norwegian and German doctors

In the German survey from 2006, the response rate for the hospital doctors was 58.2% (1,917/3,295). Of these 1,903 answered all three questions on suicidal feelings and thoughts. In the Norwegian 2010 survey, data from 528 hospital doctors was available. The gender distributions were similar in Germany and Norway, with 61.7 (52.4 to 60.8) % and 56.6 (58.9 to 64.5) % males respectively. The German doctors were significantly younger with a mean age of 39.8 (39.4 to 40.2) years vs. 47.8 (46.9 to 48.5) years. The distribution of medical specialities – with exception of internal medicine – differed between the two countries. In Norway there were fewer doctors in surgery (13.9% vs. 30.1%), gynaecology (6.3% vs. 7.1%) and anaesthesiology (7.8% vs. 14.9%) and more in psychiatry (17.8% vs. 2.7%) and other specialties (14.2% vs. 6.2%).

Norwegian hospital doctors compared with their German counterparts reported quite similar prevalences of ever felt life was not worth living (46.0, 41.8 to 50.3% vs. 39.9, 37.7 to 42.1%), ever wished to be dead (23.7, 20.1 to 27.3% vs. 24, 22.1 to 25.9%), and ever had thoughts of taking one’s life (25.9, 22.2 to 29.6% vs. 22.0, 20.1 to 23.9%).

To further explore the differences between Norwegian and German doctors, we performed multivariate logistic regressions with dichotomized versions of ever felt life was not worth living, ever wished to be dead, or ever having thoughts of taking life as response variables (“never” or “hardly ever” = 0, “sometimes” or “often” = 1). Possible predictors were country, age, gender and speciality (surgery, internal medicine, anaesthesiology, gynaecology, psychiatry and others).

Country and age were not significant predictors in any of the models. Working in psychiatry was significant in all three models: ever felt life was not worth living (OR 2.1, 95% CI 1.3 to 3.3), ever wished to be dead (2.0, 1.3 to 3.3) and ever had thoughts of taking life (2.3, 1.4 to 3.7). Being female was a significant predictor in the models of ever felt life was not worth living (1.4, 1.2 to 1.7) and ever wished to be dead (1.6, 1.3 to 2.0).

## Discussion

### Principal findings

The lifetime prevalence of ever had feelings of life not worth living, ever wished own death and ever had thoughts of taking own life among Norwegian doctors decreased from 2000 to 2010. In 2010, significant predictors of serious suicidal thoughts were lower subjective well-being, poor or average self-rated health and high psychosocial work stress, but not age, gender, speciality or job satisfaction.

### Strengths and limitations

The main strength of our study lies first and foremost in the representative dataset, making the results generalizable to the entire population of doctors in Norway. The longitudinal design is also a strength. Similarities in survey methods and comparable items from Paykel’s instrument, [6] demographics and speciality should also be pointed out. The response rates were fairly good, 86% in 2000 and 67% in 2010. This is higher than in a number of other doctor studies, [16,18,32] but do not rule out non-respondents bias.

There is of course the possibility of non-responding doctors having a lower prevalence of suicidal feelings. In one study from the US the non-respondents did not have suicidal ideation during the prior twelve months [18]. In our 2010 sample, we found no significant differences between doctors who answered in 2000 and those who did not in reporting serious suicidal thoughts, suggesting no non-respondent bias (Table 3).

That lifetime prevalence estimates of suicidal feelings are not consistent over time funds support in the literature [33]–[35]. We have shown that some doctors reported suicidal feelings ever in 2000, but never in 2010. For instance, 43.3% (91/210) of the doctors with suicidal thoughts in 2000 reported no suicidal thoughts ever in 2010 (Figure 1). To further explore possible recall bias in our study, we performed analyses on the 1.3% (16/1,253) doctors reporting one or more suicidal attempts ever in the survey in 2000. We found that four of these had since voluntarily withdrawn from the panel. Six of the remaining twelve doctors answered the question on suicidal thoughts in 2010, and two of these reported no suicidal thoughts ever. The other four reported suicidal thoughts “hardly ever” or “sometimes”. Thus, to the extent that suicidal feelings correlate with actual suicide attempts, the prevalence of suicidal feelings in our study may be underestimated due to recall bias, or other reasons for not reporting. This is important since the lifetime prevalence of suicidal thoughts is a widely used measure in psychiatric epidemiology [33].

Further limitations include the lack of items on subjective well-being, self-rated health and psychosocial work stress in the 2000 survey, and the lack of the fourth and fifth items of the Paykel’s instrument [6] in 2010, which might further elucidate our findings. Other specific elements in doctors’ personality, mental disorders, physical illnesses, and other personal risk factors might also be useful, [3,16,18,36] but such data were not available for present study.

Because the prevalence of suicidal thoughts varies between cultures, [13] and the number of foreign doctors in Norway is increasing, [36] it is also important to include this perspective in further research on suicidal ideation and thoughts of doctors.

### Comparison with other studies

Differences in methodology limit direct comparisons with other studies. However, it is possible to point out some general tendencies in suicidal thoughts.

Compared with another study among Norwegian doctors in 1993, [20] doctors in our 2000 and 2010 samples reported lower prevalence of suicidal thoughts ever: 36.3 (31.4 to 41.2) % in 1993, 30.8 (28.2 to 33.4) % in 2000 and 25.4 (22.7 to 28.2) % in 2010 (Table 2). Since the 95% confidence intervals in 1993 and 2010 do not overlap, the decrease over this 17 year period is statistically significant. Compared with data on suicidal thoughts among Norwegian police officers in 2000 [21] (22.6%, 21.1 to 24.2) and operational ambulance personnel in 2005, [22] (22.8%, 20.4 to 25.2), the doctors seem to converge with these other groups.

US surgeons in 2008 had significantly fewer cases of ever having thoughts of taking one’s own life, with 14.9 (14.1 to 15.7) % [16]. In contrast, suicidal ideation or suicidal thoughts reported by German hospital doctors in 2006 were similar to our 2010 findings.

It is challenging to compare the risk factors for serious suicidal thoughts (Table 3), because the definitions of risk group and the methods of analysis vary considerably between studies. However, our results seem to be in line with earlier studies showing that doctors with job-related stress, [3,17,19] low quality of life [18] and subjective mental or physical health complaints [3,16,18,20,37] were more at risk for reporting suicidal thoughts, while job satisfaction or age [20] had no effect. While female doctors in a previous study were more likely to have experienced serious suicidal feelings, [20] we found no such differences. There are also other studies reporting gender similarities [3,16,23]. In a study with data from 1993, anaesthesiologists had higher risk for serious suicidal thoughts [20]. In our multivariate models with ever having suicidal ideation or suicidal thought as response variables, psychiatry was a significant predictor, while in the model with ever having serious suicidal thoughts, there was no difference between medical specialties, suggesting that seriously thinking on suicide is less dependent on specialty.

### Explanation of results

The decreasing trend in suicidal feelings among Norwegian doctors from 2000 to 2010 may reflect a number of factors. The doctors who answered both in 2000 and 2010 have of course grown ten years older, from mean age 42.6 to 52.6 years. Literature suggests that mental well-being is U-shaped over the life course among Europeans, with a minimum in the mid-40s [38]. However, the observed reduction in the lifetime prevalence of suicidal feelings is approximately the same in the cohort that grew 10 years as in the two cross-sectional surveys in 2000 and 2010, where both groups are age representative (Table 2).

Another possible explanation for the changes in reporting suicidal feelings might be recall bias [33]–[35]. Doctors may consciously or unconsciously forget such details from the past (Figure 1).

There is a relationship between mental health and work conditions such as workload, stress and control over work [3,17,19,27,39]. Thus, a further reason for a decreasing trend in suicidal feelings among Norwegian doctors might lie in their work conditions. Some health care reforms have been implemented during the last decade in Norway [40]. The reforms were, at least in certain groups, often met with fear of declining professional autonomy [40,41]. However, studies with data from the last decade show that Norwegian doctors have enjoyed a stable and high level of life satisfaction, [42,43] high and increasing level of job satisfaction [40,43] and stable weekly working hours [44,45]. The fraction who perceive their workload as unacceptable is relatively small and has not increased significantly among junior doctors (19.2% in 2000, 18.5% in 2008) or consultants (26.8% in 2000, 32.2% in 2008), [44] and is actually reduced significantly among general practitioners (38.1% in 2000, 25.5% in 2008) [45]. It is possible that these trends could have a bearing on the marginal reduction in reporting suicidal feelings among Norwegian doctors.

High levels of stress and mental health disorders have been associated directly or indirectly with unfavourable lifestyle like low physical inactivity, [46] smoking [47] and heavy drinking [48]. It has also been documented that heavy drinking increases suicide risk [49]. We have recently shown that the drinking patterns of Norwegian doctors have changed from 2000 to 2010 towards more moderate alcohol consumption, fewer episodes of heavy drinking and less alcohol-related problems [50]. We have also shown that smoking decreased from 2000 to 2010, [44] and that physical activity increased in the period 1993–2010 [51]. A study based on mortality data from 1960 to 2000 documents that doctors, compared with other population groups, had lower mortality from all causes, including lifestyle-related diseases, only with the exception of suicide [1]. However, another Norwegian study suggests that the decrease in suicide rate among female doctors from 1960 to 1989 follows the general population trend [52]. Other studies from Norway, England and Wales suggest that the differences between doctors and population in suicide rates are receding [2,53]. The further development of suicide remains to be documented. In our study, ever having suicidal thoughts was approximately as common among Norwegian doctors in 2010 as among other human Norwegian service professionals like police officers and ambulance personnel.

In 2010, 7.7% of doctors had sometimes or often had thoughts of taking their own life (Table 2). Unfortunately, we do not have data on whether serious suicidal thoughts were mainly attributed to personal, social, familial or professional factors. We have shown that both work-related factors, like high psychosocial work stress and poor self-reported health and low life satisfaction were associated with serious suicidal thoughts (Table 3), suggesting an unfortunate combination of several factors. However, work-related factors might be less important that what was found in previous studies on doctors, [20] police officers [21] and ambulance personal [22] in Norway. There is also evidence that German doctors, compared with their colleagues in Norway, had lower job satisfaction, longer working hours and higher work-related stress [30,54,55]. In the sample of German and Norwegian doctors, country was not a significant predictor in any of the multivariate regression models with ever having suicidal ideation or ever having suicidal thoughts as response variables, suggesting a low importance of cultural factors, at least between these two countries.

### Policy implications

In terms of health care policy, regular preventive screening for mental health problems might be a possible strategy. Use of period prevalence through longitudinal follow-up rather than lifetime prevalence might increase the validity of the screening [33]. A recent study among university hospital doctors in Sweden and Italy shows that the majority of doctors with signs of psychological distress (78.3%) or with recent suicidal thoughts (106 of 155) did never seek professional help [56]. Since self-treatment for mental problems among doctors is common, [57] those with suicidal thoughts should be encouraged to seek psychiatric help. In Norway, initiatives financed by the Sickness Compensation Fund for Doctors (SOP) provide special programs for doctors with mental health problems [57]. Since psychosocial work stress is positively associated with serious suicidal thoughts for doctors, as shown in this study, their work organisations should constantly aim for a reduction in such stressors.

## Conclusion

The lifetime prevalence of ever had feelings of life not worth living, ever wished own death and ever had thoughts of taking own life among Norwegian doctors decreased from 2000 to 2010, suggesting decrease in suicidal risk. Compared with other professionals in Norway and doctors in Germany, Norwegian doctors showed no higher risk of suicidal thoughts in 2010. In 2010, individual and work-related factors were associated with serious suicidal thoughts among Norwegian doctors.

## Competing interest

None of the authors have any conflict of interests to declare.

## Authors’ contributions

JR and OGA contributed to concept and design of the study, analysis and interpretation of the data, and writing the article. Both authors read and approved the final manuscript.

## Pre-publication history

The pre-publication history for this paper can be accessed here:

http://www.biomedcentral.com/1471-244X/13/322/prepub
