# Correction: changes in the lifetime prevalence of suicidal feelings and thoughts among Norwegian doctors from 2000 to 2010: a longitudinal study based on national samples

**DOI:** 10.1186/1471-244X-14-1

**Published:** 2014-01-02

**Authors:** Judith Rosta, Olaf G Aasland

**Affiliations:** 1 Institute for Studies of the Medical Profession (LEFO), Norwegian Medical Association, PO Box 1152 Sentrum, 0107 Oslo, Norway; 2 Institute of Health and Society, Department of Health Management and Health Economics, University of Oslo, Oslo, Norway

## Correction

After the publication of this work [1], we became aware of some typing errors. The correct spelling of the item 3 on page 3 was “Have you ever thought of taking your own life, even if you would not really do it?”, and not “Have you ever through of taking your own life, even if you would not really do it?” The correct typing of the sentence on page 6, line 1 was “never” = 0, “hardly ever”, “sometimes” or “often” = 1, and not “never” or “hardly ever” = 0, “sometimes” or “often” = 1. We apologise for any inconvenience these may have caused.

## Pre-publication history

The pre-publication history for this paper can be accessed here:

http://www.biomedcentral.com/1471-244X/14/1/prepub
